# Identification, characterization, and expression profiling of the putative *U-box* E3 ubiquitin ligase gene family in *Sorghum bicolor*

**DOI:** 10.3389/fmicb.2022.942302

**Published:** 2022-09-15

**Authors:** Yuanpeng Fang, Qiaoli Du, Qian Yang, Junmei Jiang, Xiaolong Hou, Zaifu Yang, Degang Zhao, Xiangyang Li, Xin Xie

**Affiliations:** ^1^Key Laboratory of Agricultural Microbiology, College of Agriculture, Guizhou University, Guiyang, China; ^2^State Key Laboratory Breeding Base of Green Pesticide and Agricultural Bioengineering, Key Laboratory of Green Pesticide and Agricultural Bioengineering, Ministry of Education, Guizhou University, Guiyang, China; ^3^College of Life Sciences, Ministry of Education, Institute of Agricultural Bioengineering, Key Laboratory of Mountain Plant Resources Protection and Germplasm Innovation, Guizhou University, Guiyang, China; ^4^Guizhou Academy of Agricultural Sciences, Guizhou Conservation Technology Application Engineering Research Center, Guizhou Institute of Prataculture, Guizhou Institute of Biotechnology, Guiyang, China

**Keywords:** *U-box*, expression pattern, abiotic stress, co-regulatory networks, *Sorghum bicolor*

## Abstract

The *U-box* family is one of the main E3 ubiquitin ligase families in plants. The *U-box* family has been characterized in several species. However, genome-wide gene identification and expression profiling of the *U-box* family in response to abiotic stress in *Sorghum bicolor* remain unclear. In this study, we broadly identified 68 *U-box* genes in the sorghum genome, including 2 *CHIP* genes, and 1 typical *UFD2* (Ub fusion degradation 2) gene. The *U-box* gene family was divided into eight subclasses based on homology and conserved domain characteristics. Evolutionary analysis identified 14, 66, and 82 *U-box* collinear gene pairs in sorghum compared with arabidopsis, rice, and maize, respectively, and a unique tandem repeat pair (*SbPUB26*/*SbPUB27*) is present in the sorghum genome. Gene Ontology (GO) enrichment analysis showed that U-box proteins were mainly related to ubiquitination and modification, and various stress responses. Comprehensive analysis of promoters, expression profiling, and gene co-regulation networks also revealed that many sorghum *U-box* genes may be correlated with multiple stress responses. In summary, our results showed that sorghum contains 68 *U-box* genes, which may be involved in multiple abiotic stress responses. The findings will support future gene functional studies related to ubiquitination in sorghum.

## Introduction

Sorghum (*Sorghum bicolor*) is used in winemaking, bioenergy, and feed plant industries worldwide, and is the most important winemaking crop in China (Mace et al., 2013). Since the completion of genome sequencing in 2013 (Mace et al., 2013), sorghum breeding based on genome research has expanded rapidly (Vallabhaneni et al., 2010; Zhao et al., 2011; Yang and Wang, 2016; Ding et al., 2019; Yang et al., 2020). However, biological (*Colletotrichum graminicola* and *Mythimna separata* infection, etc.) and non-biological stresses (drought, hormonal, salt stress, etc.) affect the quality and quantity of this important crop (Sharma, 1993; Anaso, 2010; Silva et al., 2013, 2021; Dowd et al., 2016; Cuevas et al., 2018; Das et al., 2019; Moharam and Hassan, 2020). Previous studies have shown that epigenetic modification proteins are related to plant stress resistance, providing a potential resource for plant resistance breeding (Callis, 2014; Yang et al., 2018; Miryeganeh and Saze, 2020; Yu et al., 2021; Puyuan et al., 2022).

Epigenetic regulation involves site-specific modification of nucleic acids and proteins under environmental stress, and it occurs widely in all animals, plants, and microorganisms. The epigenetic modification mechanism mainly includes pre-translational modification (DNA modification, non-coding RNA regulation, and chromatin remodeling) and post-translational modification (histone modification and non-histone modification). Post-translational modification (PTM), an important type of epigenetic regulation, mainly includes protein methylation, acetylation, ubiquitination, phosphorylation, etc. (Miryeganeh and Saze, 2020). Methylation and acetylation are generally considered to regulate protein activity, while phosphorylation initiates kinase activity (Yu et al., 2021). By contrast, ubiquitination mainly mediates protein clearance and recycling process by promoting the stability of active proteins and the repair of misfolded proteins to achieve protein equilibrium (Callis, 2014). Reports showed that ubiquitination plays principal roles in the regulation of plant stress resistance, for example, abscisic acid (ABA)-insensitive RING protein (AIRP) in some maize cultivars is more sensitive to ABA than wild varieties, thus achieving higher drought tolerance (Kong et al., 2015; Yang et al., 2018). The ubiquitin ligase TagW2-6a negatively regulates gibberellin (GA) synthesis and signaling (An et al., 2021). E3 ligase MIEL1 negatively regulates jasmonic acid (JA) signaling and thereby reduces cold resistance in apple (*Malus domestica*) by mediating ubiquitination and degradation of the novel B-box (BBX) protein BBX37 (You and He, 2016). Besides, studies also have shown that ubiquitination is a potential resource for plant disease resistance.

Ubiquitination modifications include conventional ubiquitination modification (with ubiquitin as the substrate) and SUMO-type ubiquitination modification (with the ubiquitin-like SUMO molecule as the substrate) (Zhou and Zeng, 2017). Conventional ubiquitination typically involves defined steps: (1) first, E1 ubiquitin activase (UBA) activates ubiquitin in the presence of ATP, allowing the cysteine residues of UBA to form the thioester-linked intermediate E1-ubiquitin (E1-Ub) (Mandal et al., 2018); (2) subsequently, the E2 ubiquitin-binding enzyme (UBC) interacts with UAB-Ub and transfers activated Ub to an active cysteine residue of UBC to form a thioester-linked UBC-Ub intermediate (Mandal et al., 2018); (3) finally, the ubiquitin ligase (E3) interacts with the target protein and E2-Ub to create an isopeptide bond between the C-terminal glycine residue and the lysine residue of Ub. The establishment of an isopeptide bond between this glycine residue and the lysine residue of the target protein results in the transfer of Ub to the target protein by ubiquitin ligase (E3) (Mandal et al., 2018). The ubiquitination modification system includes a large protein family, with ∼10 E1 ubiquitin activating enzymes, ∼50 E2 ubiquitin conjugating enzymes, and ∼400 E3 ubiquitin ligases in plants (Richard, 2009). In general, the ubiquitination modification pathway includes three important factors; E1 ubiquitin-activating enzyme (UAE), E2 ubiquitin conjugating enzyme (UBC), and E3 ubiquitin ligases, including RING, U-box, HECT (homologous to the E6-AP carboxy terminus), and CRL (cullin-ring ubiquitin ligase) (Richard, 2009). Among them, E3 ubiquitin ligase is the most abundant, and it also influences ubiquitinated substrate diversity (Rennie et al., 2020). The numerous substrate-selective E3 ubiquitin ligases are mainly classified into RING-finger, HECT, and U-box structural domain classes (Mandal et al., 2018). Due to their specific non-spontaneous ubiquitination activity and their ability to participate in the degradation of unfolded or misfolded proteins upon activation by cofactors, U-box-like proteins are important, but they are complex and more difficult to control than other E3 ubiquitin ligases. Therefore, clarifying the unique contributions of different U-box proteins to different stress processes will help us to understand the onset of plant resistance (Li et al., 2017; Kim et al., 2021; Tang et al., 2021).

Most *U-box* gene family members contain U-box domains, and these main ligases control the conventional ubiquitination modification process that involves attaching ubiquitin to substrates, thereby altering multiple protein activities (Azevedo et al., 2001; Hu et al., 2018; Tong et al., 2021). Specifically, U-box type E3 obtains ubiquitin from E2 through the conserved U-box domain, through salt bridges, ion chelation, and non-covalent hydrogen bonding interactions, and passes it to target proteins. In previous studies, cotton *PUBs* and banana *U-box* genes were found to be widely responsive to abiotic stress (Hu et al., 2018; Lu et al., 2020). Examples include *OsPUB67* that can enhance tolerance to drought stress by regulating ABA, thereby enhancing the capacity to scavenge reactive oxygen species (ROS) and stomatal closure capacity (Qin et al., 2020). AtPUB11 degrades AtLRR1 (LEUCINE RICH REPEAT PROTEIN 1) and Atkin7 (KINASE 7), and negatively regulates ABA-mediated drought tolerance in *Arabidopsis thaliana* (Chen X. X. et al., 2020). However, *U-box* genes have important value for plant resistance, and they have been identified in rice (*Oryza sativa*), tomato (*Solanum lycopersicum*), apple (*Malus domestica*), banana (*Musa nana*), soybean (*Glycine max*), Chinese cabbage (*Brassica rapa*), cotton (*Gossypium hirsutum*), and cabbage (*Brassica oleracea*) (Azevedo et al., 2001; Li et al., 2009; Wang et al., 2015, Wang J. et al., 2020; Wang K. L. et al., 2020; Hu et al., 2018, 2019; Sharma and Taganna, 2020). However, comprehensive genome-wide identification of *U-box* genes in sorghum has not been performed, and expression characteristics have not been analyzed.

In this study, 68 *U-box* genes were identified in sorghum, and phylogenetic relationships, conserved structures, gene structures, and collinearity were systematically analyzed by bioinformatics. In addition, tissue-specific and stress-related expression patterns were analyzed by RNA sequencing (RNA-seq) under GA, MeJA (methyl jasmonate), ABA, and PEG (polyethylene glycol) 6000 treatments, and the results were validated by real-time quantitative PCR (RT-qPCR). The findings provide a basis for further functional exploration of *U-box* genes in sorghum resistance breeding, and shed new light on the molecular evolution of the *U-box* gene family.

## Materials and methods

### Plant materials, growth conditions, and stress treatments

Sorghum (BTx623) seeds were obtained from the Key Laboratory of Agricultural Microbiology, College of Agriculture, Guizhou University, Guiyang, China. After surface disinfection, the sorghum BTx623 seeds were soaked in sterilized water for 24 h and then moisturized with gauze for 72 h. The germinated seeds (25°C) were planted in sterilized nutrient soil (PINDSTRUP, Denmark) and cultivated in a greenhouse with a relative humidity of 75% at 25/20°C under a 14 h light/10 h dark cycle. In the three-leaf stage, ABA (200 μM), 20% PEG 6000, GA (100 μM), and MeJA (100 mM) were sprayed onto the whole plants in seedlings separately (Townsley et al., 2013; Chai and Subudhi, 2016; Yimer et al., 2018; Miao et al., 2019; Zeng et al., 2019). Sorghum leaves were collected at 0, 3, 6, 9, and 12 h after treatment. All samples included three biological replicates, with four seedlings treated per replicate. All samples were immediately frozen in liquid nitrogen after sampling and stored at −80°C until RNA extraction (Wu et al., 2019).

### Total RNA extraction and real-time quantitative PCR analysis

Total RNA was extracted using TRIzol reagent (Thermo Fisher, United States), and the cDNA was synthesized using HiScript III RT SuperMix (Vazyme Biotech, Beijing, China) (Wu et al., 2019). Real-time fluorescent quantitative PCR (RT-qPCR) was used to determine gene expression patterns. The reaction system was composed of 4.5 μL of cDNA, 7.5 μL of SYBR mix, 0.3 μL of each primer, and 15 μL of ddH_2_O. Thermal cycling included 40 cycles at 95°C for 5 min, 95°C for 10 s, and 58°C for 30 s, and one cycle at 95°C for 10 s, 58°C for 60 s, and 95°C for 10 s. Three biological replicates were performed, and *SbEIF4a* was used as an internal reference gene (Yang et al., 2020; Du et al., 2021; Jin et al., 2021). RT-qPCR data were analyzed using the 2^–Δ^ ^Δ^ *^Ct^* method (Pfaffl, 2001), and Duncan’s new multiple range test (SPSS software) was used for significance analysis.

### Identification of the *U-box* gene family in *Sorghum bicolor*

To identify members of the sorghum *U-box* gene family, the basic conserved domain (U-box: PF04564) was searched against EnsemblPlant^1^ using HMMER 3.6.1 software (*E* value limited to 0.05, other parameters remained as default values) (Potter et al., 2018), and domain confirmation was carried out using PFAM and SMART (Letunic and Bork, 2018; El-Gebali et al., 2019). Conserved motifs were predicted using MEME^2^ and 20 motifs were explored (*E* value limited to 0.05, other parameters remained as default values). Chromosome distribution, conserved motifs, and gene structure visualization were carried out using TBtools 1.0 (Chen C. et al., 2020). Analysis of physicochemical properties was performed by ExPASy^3^ (Bailey et al., 2009; Artimo et al., 2012; Chen C. et al., 2020). Gene naming uses a combination of homology and sequence naming. The *PUB*, *CHIP*, and *UFD* genes among *U-box* members were first identified through homology, then genes were named sequentially based on chromosome positioning.

### Evolutionary analysis of *U-box* genes

To explore the evolutionary relationships and subcategories of *U-box* genes between sorghum and arabidopsis, MEGA 7.0 software (Kumar et al., 2016) was used to construct homologous protein rootless evolutionary trees *via* the neighbor-joining (NJ) method with 1,000 bootstrap replicates, followed by ITOL.^4^ Selection of tandem repeat gene pairs in sorghum was carried out with (a) short sequences covering 75% and (b) aligned regions of longer genes with 75% similarity (Gu et al., 2002). Whole-genome replication examination was performed by MCScan X (Wang et al., 2013). OrthoVenn2^5^ was used to query homologous genes in different plants (Xu et al., 2019).

### Characteristics of *U-box* genes’ promoter and gene ontology annotation

AgriGO^6^ (Tian et al., 2017) and AIpuFu^7^ were employed to investigate the main functions and Gene Ontology (GO) annotation features of sorghum *U-box* genes. To explore the promoter and expression characteristics of sorghum *U-box* genes, putative promoter sequences were extracted (sequences 2 kb before the 5′ end of the sequence) using TBtools1.0 (Chen C. et al., 2020). Cis-acting elements were predicted by PlantCARE^8^ and plotted by TBtools1.0 (Lescot et al., 2002; Chen et al., 2020).

### Expression profiling analysis of *U-box* gene

To analyze the expression profiling of sorghum *U-box* genes in different tissues, and under drought stress and osmotic stress, an FPKM (fragments per kilobase of exon per million fragments mapped) expression matrix provided by the Sorghum functional database (Supplementary Table 5) (Tian et al., 2016) and SRA^9^ (PRJDB1973 and PRJDB1973) was extracted and mapped by TBtools1.0 (Chen C. et al., 2020).

### Co-regulatory network analysis of *U-box* genes

Arabidopsis FPKM data were downloaded from the Expression Atlas website^10^ for SAMN01041946 and SAMN02440041. Based on the previously obtained expression values in FPKM format for arabidopsis and sorghum *U-box* genes, Pearson correlation coefficients (PCC) and *p*-values were calculated to obtain the expression levels of *U-box* genes by the Pearson method using R software. Correlation heat maps were generated by the corrplot tool in R software (version 0.84). Gene co-regulatory networks were constructed by Cytoscape version 3.7.1 (Shannon et al., 2003) based on the PCCs of *U-box* gene pairs with a *p*-value ≤ 0.05 (Zhao et al., 2019).

## Results

### Genome-wide identification of sorghum *U-box* family members

In this study, a total of 68 *U-box* genes with complete U-box domains, including a typical ubiquitin fusion degradation 2 (*UFD2*) gene, two carboxyl terminus of heat shock protein 70-interacting protein (*CHIP*) genes, and 65 plant U-box (*PUB*) genes were identified in sorghum genome by using hidden Markov model (HMMER) method (Table 1; Potter et al., 2018).

**TABLE 1:** Basic information of sorghum U-box genes.

Gene name	Gene ID	Chromosome	Start	End	CDS length	Protein length	Molecule weight (Da)	pI (protein isoelectric point)
*SbPUB1*	SORBI_3001G226000	1	21,606,961	21,616,792	1,581	526	57,608.9	6.51
*SbPUB2*	SORBI_3001G272900	1	52,754,506	52,755,990	1,485	494	51,909.8	5.96
*SbPUB3*	SORBI_3001G305800	1	58,991,748	58,995,101	1,296	431	48,599.6	4.99
*SbPUB4*	SORBI_3001G306000	1	59,003,709	59,006,974	1,779	592	67,199.1	6.19
*SbUFD2*	SORBI_3001G306300	1	62,162,915	62,172,033	1,500	499	55,776.9	5.96
*SbPUB5*	SORBI_3001G333100	1	62,300,389	62,308,556	3,093	1,030	115,815.5	5.1
*SbPUB6*	SORBI_3001G334400	1	70,150,749	70,155,949	2,004	667	76,061.8	6.15
*SbPUB7*	SORBI_3001G420500	1	71,899,741	71,901,707	2,250	749	81,188	4.99
*SbPUB8*	SORBI_3001G441100	1	72,598,605	72,601,731	1,365	454	48,019	8.16
*SbPUB9*	SORBI_3001G448700	1	74,000,494	74,004,078	1,380	459	49,388.1	7.57
*SbPUB10*	SORBI_3001G466700	2	20,708,312	20,713,727	1,776	591	65,030.1	7.84
*SbPUB11*	SORBI_3002G136800	2	20,854,345	20,857,919	2,178	725	81,104.2	6.4
*SbPUB12*	SORBI_3002G137000	2	57,447,925	57,451,045	1,857	618	70,360.5	6.98
*SbPUB13*	SORBI_3002G188700	2	57,860,760	57,862,523	2,151	716	75,503.6	7.77
*SbPUB14*	SORBI_3002G192101	2	58,593,486	58,602,167	1,764	587	60,276.4	6.77
*SbPUB15*	SORBI_3002G196800	2	66,883,033	66,891,707	2,592	863	94,918.3	7.07
*SbPUB16*	SORBI_3002G290500	3	709,579	717,183	2,466	821	90,627.1	6.35
*SbPUB17*	SORBI_3003G008100	3	66,235,870	66,239,678	4,215	1,404	150,368.3	6.15
*SbPUB18*	SORBI_3003G339500	3	68,325,094	68,326,611	2,109	702	74,999.2	6.05
*SbPUB19*	SORBI_3003G367000	3	69,362,960	69,366,951	1,227	408	43,422.6	8.58
*SbPUB20*	SORBI_3003G379800	3	70,246,609	70,251,164	2,418	805	87,934.2	5.38
*SbPUB21*	SORBI_3003G391300	4	7,929,697	7,934,215	2,409	802	87,816.7	6.22
*SbPUB22*	SORBI_3004G093100	4	9,697,392	9,699,581	1,845	614	69,105.6	6.28
*SbPUB23*	SORBI_3004G103000	4	45,719,715	45,724,433	2,190	729	78,826.8	8.32
*SbPUB24*	SORBI_3004G146800	4	46,354,343	46,359,187	1,395	464	50,761.2	6.3
*SbPUB25*	SORBI_3004G147600	4	46,930,572	46,935,381	1,827	608	66,991.5	6.37
*SbPUB26*	SORBI_3004G149466	4	52,003,635	52,005,512	1,908	635	71,346.2	9.65
*SbPUB27*	SORBI_3004G169200	4	52,042,173	52,045,045	1,284	427	46,070.6	8.55
*SbPUB28*	SORBI_3004G169300	4	52,146,188	52,147,549	1,275	424	45,759.2	8.43
*SbPUB29*	SORBI_3004G169900	4	52,422,097	52,423,320	1,362	453	46,729.8	7.99
*SbPUB30*	SORBI_3004G171700	4	59,301,645	59,303,033	1,224	407	42,044.9	8.3
*SbPUB31*	SORBI_3004G245100	4	59,600,610	59,606,637	1,389	462	48,910	8.36
*SbPUB32*	SORBI_3004G249100	4	59,895,818	59,901,343	1,917	638	68,895.1	6.37
*SbPUB33*	SORBI_3004G253100	4	61,158,823	61,164,858	2,898	965	106,627.1	6.57
*SbPUB34*	SORBI_3004G267300	4	61,914,064	61,915,353	2,319	772	86,704.1	5.38
*SbPUB35*	SORBI_3004G275800	4	63,031,840	63,038,340	1,290	429	46,088.9	5.19
*SbPUB36*	SORBI_3004G288900	4	63,040,890	63,045,569	3,117	1,038	117,737.8	6.31
*SbPUB37*	SORBI_3004G289000	4	67,964,339	67,968,893	2,202	733	81,498.7	6.17
*SbPUB38*	SORBI_3004G350900	4	68,530,605	68,533,824	2,295	764	83,129.3	6.18
*SbPUB39*	SORBI_3004G358700	6	29,537,324	29,539,918	2,547	848	92,039.5	5.36
*SbPUB40*	SORBI_3006G042500	6	43,206,592	43,207,420	2,034	677	74,414	6.32
*SbPUB41*	SORBI_3006G075400	6	43,950,060	43,951,792	1,389	462	48,837.6	8.44
*SbPUB42*	SORBI_3006G075801	6	44,041,315	44,043,956	1,344	447	46,602.7	8.16
*SbPUB43*	SORBI_3006G086400	6	45,581,596	45,584,458	1,197	398	41,742.7	8.51
*SbPUB44*	SORBI_3006G086700	6	45,602,508	45,607,149	2,463	820	93,167.3	6.71
*SbPUB45*	SORBI_3006G127800	6	49,275,639	49,280,945	2,568	855	93,781.5	5.11
*SbPUB46*	SORBI_3006G192400	6	54,626,363	54,631,210	2,979	992	108,121.7	6.16
*SbPUB47*	SORBI_3006G196400	6	54,919,919	54,921,554	1,239	412	44,709.5	8.15
*SbPUB48*	SORBI_3006G274933	6	60,616,840	60,619,003	1,281	426	45,485.6	8.04
*SbPUB49*	SORBI_3007G011400	7	1,025,117	1,032,828	3,024	1,007	108,219.7	6.45
*SbCHIP1*	SORBI_3007G014900	7	1,299,601	1,304,286	828	275	30,873.9	6.62
*SbPUB50*	SORBI_3007G034600	7	3,027,546	3,028,613	1,068	355	36,931.8	6.76
*SbPUB51*	SORBI_3007G126300	7	53,898,592	53,901,103	2,145	714	75,022.2	7.47
*SbPUB52*	SORBI_3007G130100	7	54,683,055	54,685,169	1,722	573	59,195.2	7.73
*SbPUB53*	SORBI_3007G226500	7	65,359,657	65,361,668	1,653	550	60,531.6	7.88
*SbPUB54*	SORBI_3008G044300	8	4,362,514	4,363,878	1,365	454	47,729.3	6.66
*SbPUB55*	SORBI_3008G089100	8	31,663,361	31,667,580	1,893	630	69,645	5.05
*SbPUB56*	SORBI_3008G136200	8	56,500,309	56,502,578	1,560	519	56,108.7	5.34
*SbCHIP2*	SORBI_3009G002800	9	224,225	227,090	837	278	31,515.7	6.09
*SbPUB57*	SORBI_3009G153200	9	50,944,346	50,947,868	1,374	457	46,859.6	8.21
*SbPUB58*	SORBI_3009G173300	9	52,836,152	52,838,191	2,040	679	71,538.9	6.93
*SbPUB59*	SORBI_3010G000900	10	83,990	86,626	1,806	601	64,961.9	7.34
*SbPUB60*	SORBI_3010G030300	10	2,467,287	2,473,282	2,424	807	89,529.9	6.68
*SbPUB61*	SORBI_3010G050700	10	3,919,877	3,924,808	1,983	660	74,696.4	6.02
*SbPUB62*	SORBI_3010G102000	10	9,458,742	9,460,121	1,380	459	47,720.3	8.82
*SbPUB63*	SORBI_3010G140500	10	27,155,811	27,185,775	2,223	740	82,608	7
*SbPUB64*	SORBI_3010G174500	10	51,011,401	51,016,873	2,352	783	87,335.1	7.11
*SbPUB65*	SORBI_3010G274100	10	60,713,126	60,717,604	1,893	630	69,861	6.04

Sorghum *U-box* genes were distributed in all chromosomes except chromosome 5, with *CHIP* genes on chromosomes 7 and 9, and the *UFD2* gene on chromosome 1. The number of amino acids per protein ranges from 400 to 1,500, and the relative molecular weight of Sorghum PUB proteins is 40–160 kDa. Sorghum PUB5, PUB17, PUB33, PUB36, PUB46, and PUB49 are among the largest, with relative molecular weights >100 kDa. By contrast, the Sorghum CHIP proteins are relatively small with the protein weight between 30 and 40 kDa. The isoelectric point (pI) ranges from 4.99 to 9.65, from medium strong acid to medium strong base. SbPUB26 has the highest pI and SbPUB3/7 have the lowest pI. UFD2 and CHIP proteins have pI values in the weakly acidic range (Table 1). These indicated that sorghum U-box proteins are divergent in protein characteristics.

**FIGURE 1:**
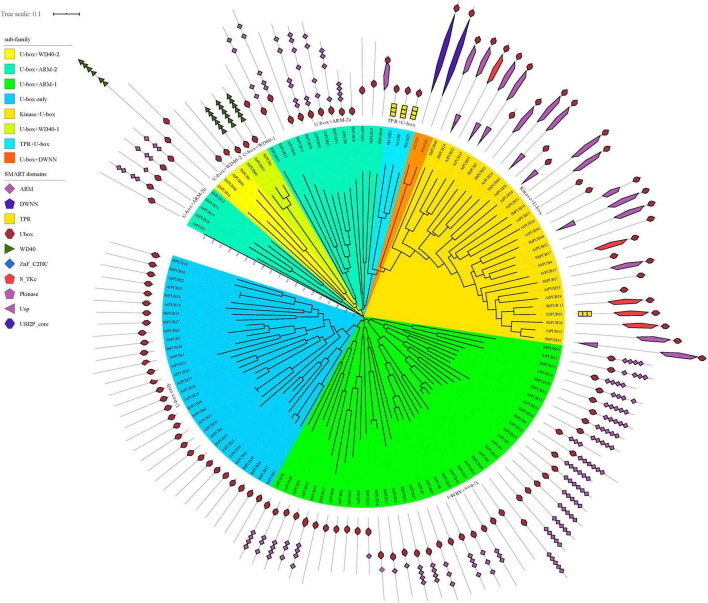
Phylogenetic tree and domain analysis of *U-box* genes in Sorghum and *Arabidopsis*. Phylogenetic relationships between *U-box* genes in *Sorghum bicolor* and *Arabidopsis thaliana* were explored. Rootless trees were generated by the neighbor-joining (NJ) method using MEGA7 software. Numbers next to branches indicate 1,000 bootstrap replicates as a percentage. The phylogenetic classification of *U-box* genes is marked with corresponding colors and different shapes. Colors in the outer ring represent different domains, as shown in the color legend in the upper left corner. U-box, Ub fusion degradation 2 (UFD2) domain; PUB, plant U-box; UFD, Ub fusion degradation; CHIP, carboxyl terminus of heat shock protein 70-interacting protein; At, *Arabidopsis thaliana*; Sb, *Sorghum bicolor*.

Based on the phylogenetic relationships and domain composition of *A. thaliana* and sorghum *U-box* genes, they can be divided into eight categories; U-box only, Kinase + *U-box*, *U-box* + WD40-1, *U-box* + WD40-2, *U-box* + Armadillo 1 (ARM-1), *U-box* + ARM-2, *U-box* + Tetratricopeptide repeat (TPR) or CHIP, and U-box + Domain With No Name (DWNN) or UFD2 (Figure 1 and Supplementary Table 1).

The sorghum *U-box* gene family includes an extra *CHIP* gene and six *PUB* genes than that in arabidopsis. Most sorghum *U-box* genes share one ortholog in arabidopsis, as exemplified by *AtCHIP*/*SbCHIP1*, *SbUFD2*/*AtUFD2*, and *SbPUB49*/*AtPUB9*, indicating similar evolutionary pathways (Supplementary Table 2). However, some gene homology branches such as *SbPUB16*/*SbPUB46*, *SbPUB1*/*SbPUB12*, and *AtPUB18*/*AtPUB19* appear to reflect divergence shortly after the separation in these two species. Within the same category, a large number of structural changes are evident; for example, the Kinase + U-box within the *SbPUB61* branch divides into the *CHIP* branch containing genes with a TPR domain structure, and multiple branches only contain proteins with a U-box domain. Some homologous genes appear to lack intact structural domains, such as U-box + ARM-1 within the *SbPUB13* branch that contain only a U-box domain but no ARM structural domain (Figure 1). These indicated that sorghum *U-box* genes are divergent in structural domains, showing complex evolutionary pathway.

### Structures and conserved motifs of *U-box* genes in sorghum

**FIGURE 2:**
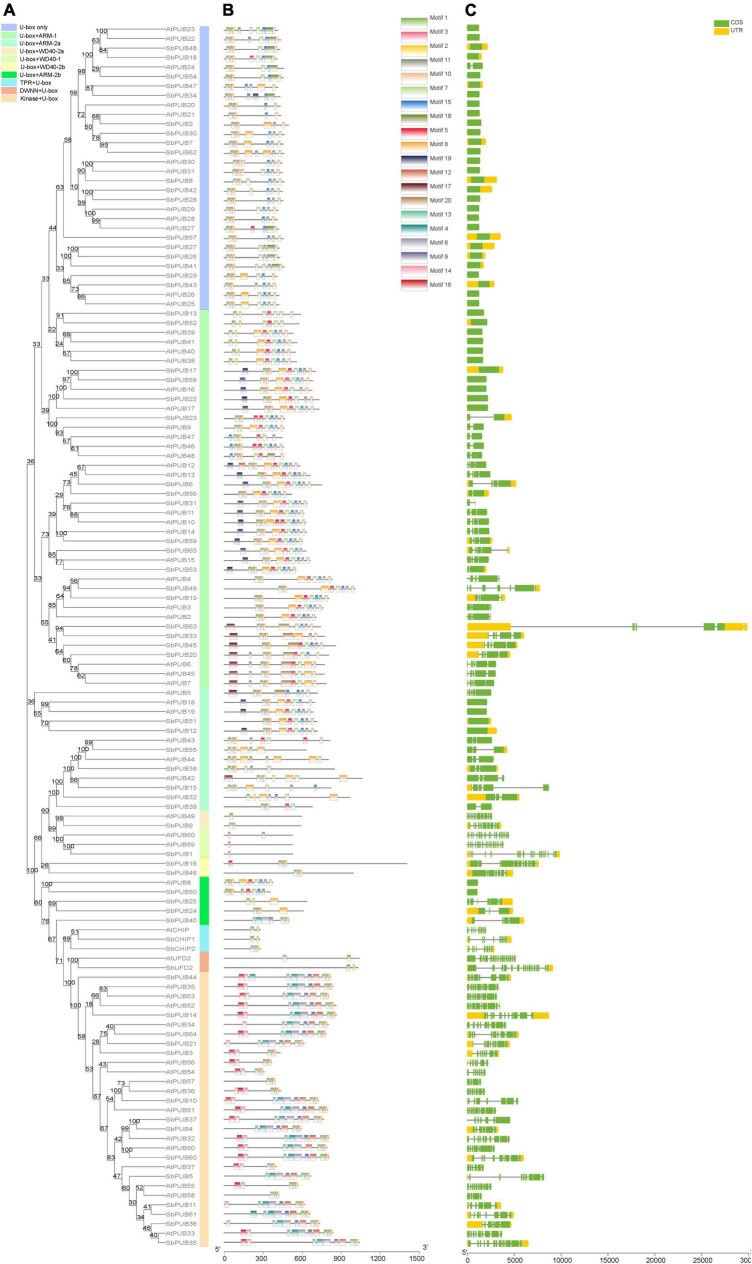
Motif and structural analysis of *U-box* gene family members in Sorghum and *Arabidopsis*. **(A)** Phylogenetic relationships between *U-box* genes of *Sorghum bicolor* and *Arabidopsis thaliana*. Rootless trees were generated by the neighbor-joining (NJ) method in MEGA7 software. Numbers next to branches indicate 1,000 bootstrap replicates as a percentage. Different background colors represent different subfamilies. **(B)** Motifs in *U-box* genes. Different colored rectangles represent 30 different motifs, as shown in the color legend in the upper right corner. **(C)** Structural analysis of *U-box* genes. Green and yellow indicate coding sequence (CDS) and untranslated region (UTR) features, respectively, and the horizontal line represents gene regions, as shown in the color legend in the upper right corner.

As shown in Figure 1, the domains of CHIP and UFD2 proteins are clearly distinguished, and the motifs of CHIP and UFD2 are also significantly different (Figures 2A,B). CHIP proteins contain at least two motifs (1/2). The UFD2 protein consists of two motifs (1). For PUBs, the distribution of their motifs is not completely conserved. Specifically, U-box + WD40-1 and U-box + WD40-2 branch proteins share the lowest similarity with PUB branch proteins with core motifs 14. Interestingly, the U-box + WD40-2 branch is classified into two categories: a typical branch SbPUB49/AtPUB9, and the sorghum-specific branch SbPUB16/SbPUB46. The U-box + ARM branch is divided to three groups: *U-box* + ARM1 (motifs 1/2/5/7), U-box + ARM2a (motifs 1/2/3), and U-box + ARM2b (motifs 1/2/3). Finally, the Kinase + *U-box* and U-box only branches include one core motifs (1) and four core motifs (1/2/4/10), respectively (Figures 2A,B).

In terms of gene structure, both *CHIP* and *UFD2* genes include a complex gene structure with >6 introns (Figures 1, 2C). Regarding the gene structure of *PUB* genes, some maintain low complexity, as exemplified by *SbPUB6* and *SbPUB23* that contain 1–5 introns, while *SbPUB1* and *SbPUB9* have high complexity (>10 introns). It is worth noting that all gene groups with a single U-box domain have a simple gene structure, mostly with 0 or 1 intron. In the groups containing both U-box and ARM domains, the corresponding gene structure is complex than that of the single U-box groups, and the number of introns varies between 0 and 5. In addition, the groups with multi-domains in the *U-box* gene (Kinase + U-box, U-box + WD40, U-box + TPR, and U-box + DWNN) have the most complex gene structure (>10 introns; Figures 2A,C).

### Chromosome localization and tandem duplication of sorghum *U-box* genes

**FIGURE 3:**
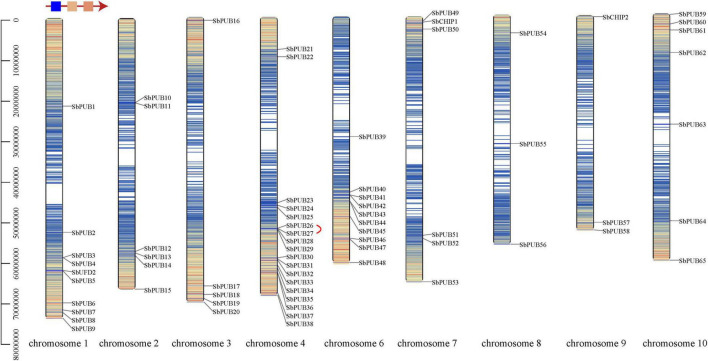
Chromosome location of *U-box* genes in Sorghum. The different colors of chromosomes represent gene density in this range, and variation is indicated in the upper left corner. Red lines represent tandem repeats.

In order to understand the chromosomal distribution and replication pattern of sorghum *U-box* genes, gene mapping and tandem duplication were analyzed to explore the diversification of *U-box* genes. Ten (containing a typical *UFD2* gene), 6, 5, 18, 0, 10, 6, and 3 (with 1 *CHIP1*), 3 (with 1 *CHIP2*), and 7 *U-box* genes were found to be distributed on chromosomes 1–10, respectively. Most *PUB* genes are distributed in the same cluster, but the two *CHIP* genes are not adjacent to each other, and are relatively distant from the *PUB* genes (Figure 3 and Table 1). Interestingly, *UFD2* genes are located in the low-density distribution region, and *CHIP* genes are present in the region with high gene density. The *CHIP* gene is near the start of the chromosome, while *UFD2* is in a region near the end of the chromosome. Most *PUB* genes also form large gene clusters near the end of chromosomes (Figure 3). Additionally, chromosome 4 includes 18 *PUB* genes, most of which form gene cluster at the end of this chromosome (Figure 3).

**FIGURE 4:**
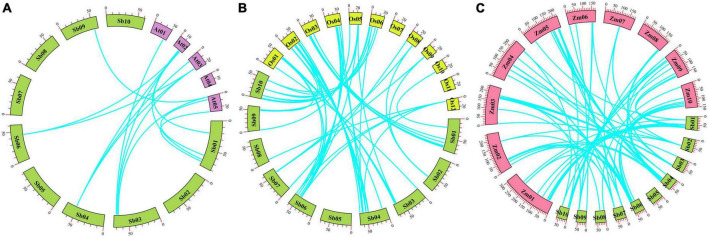
Collinear block identification of sorghum and *Arabidopsis*, rice, and maize *U-box* genes. **(A)** Collinearity relationships between *U-box* genes in sorghum and *Arabidopsis thaliana*; **(B)** collinear relationships between *U-box* genes in sorghum and rice; **(C)** collinearity relationships between *U-box* genes in sorghum and maize.

Based on a cut-off of 75% sequence similarity and coverage (Potter et al., 2018), only one tandem duplicate gene pair was found, the *SbPUB26*/*SbPUB27* gene pair, located in a region of low gene density at the end of chromosome 4 (Figure 3). Gene collinearity results showed that there are 14 collinear relationships between *S. bicolor* and *A. thaliana U-box* genes. Among them, chromosome 1 and chromosome 4 contain the largest number of genes, and the highest collinearity (Figure 4A). However, collinearity between rice, maize, and sorghum *U-box* genes was high, with 66 and 82 showing collinearity, respectively. Among them, in sorghum, chromosomes 4 and 6 include the highest collinearity gene distribution, compared with chromosome 2 in rice and chromosomes 3 and 5 in maize (Figures 4B,C). In particular, neither rice chromosome 11 nor sorghum chromosome 5 showed any collinearity of *U-box* genes. Furthermore, collinearity was observed for all types of *U-box* genes (49 sorghum *U-box* genes), but no collinearity was found for *CHIP* gene types between *S. bicolor* and *A. thaliana* (Figure 4 and Supplementary Table 3).

### Promoter and functional enrichment of sorghum *U-box* genes

**FIGURE 5:**
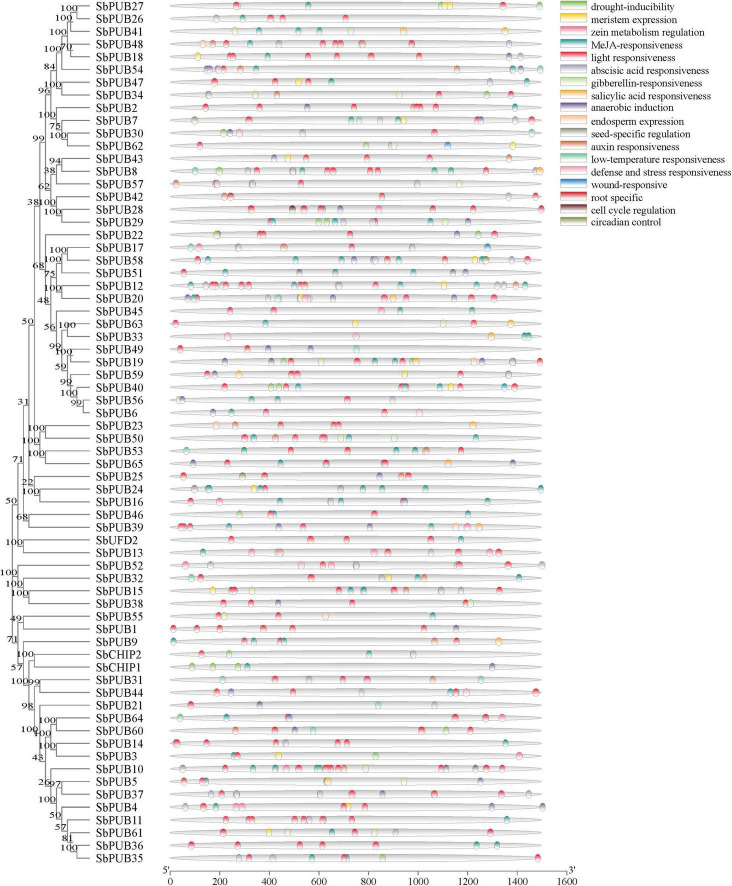
Promoter analysis of sorghum *U-box* genes. Rootless trees were generated by the neighbor-joining (NJ) method using MEGA7 software. Numbers next to branches indicate 1,000 bootstrap replicates as a percentage. Different promoters are indicated by rectangles of different colors to represent homeopathic elements. Detailed comments are included in the top right panel.

Numerous tissue-specific elements, environmental response elements, and hormone response elements were identified in sorghum *U-box* gene promoters. The promoter of *SbUFD2* contains two light response, three hormone response, and four environment response elements. The *SbCHIP* promoter contains drought response elements. Most *SbPUB* gene promoters are rich in light response elements, followed by hormone response elements such as MeJA, GA, ABA, and drought, and they also contain the environmental response or tissue-specific expression elements. Some gene promoters also include injury response elements, such as *SbPUB16* and *SbPUB62* (Figure 5).

In particular, MeJA or GA response elements occur repeatedly in *PUB* genes. For example, MeJA response element occurred three times in *SbPUB7*, *SbPUB8*, *SbPUB9*, *SbPUB11*, *SbPUB15*, *SbPUB18*, *SbPUB23*, *SbPUB28*, *SbPUB29*, *SbPUB34*, *SbPUB39*, *SbPUB41*, *SbPUB50*, *SbPUB51*, *SbPUB53*, *SbPUB58*, and *SbPUB61* promoters. Additionally, ABA or drought response elements were also found in *SbPUB10*, *SbPUB11*, *SbPUB12*, *SbPUB14*, *SbPUB19*, *SbPUB28*, *SbPUB36*, *SbPUB52*, *SbPUB57*, *SbPUB58*, *SbPUB65*, and *SbPUB7* in the sorghum genome when searching the EnsemblPlant database. Collectively, in the promoters of *SbPUB11*, *SbPUB28*, *SbPUB58*, and *SbPUB7*, three ABA and MeJA response elements were simultaneously detected, indicating that these genes may be involved in responses to stress related to JA and ABA (Figure 5).

**FIGURE 6:**
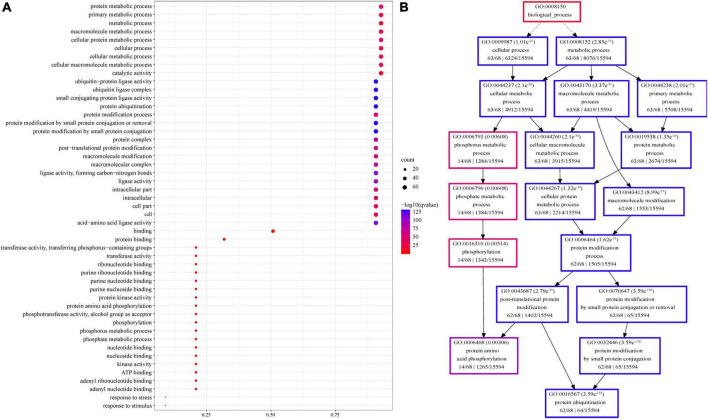
Gene Ontology enrichment of sorghum U-box members. **(A)** GO enrichment presented as dot bubbles. **(B)** Corresponding pathway diagram of enriched GO terms. GO enrichment was conducted by Agrigo, and corrected *p*-values were calculated by R version 3.6.2.

In addition, GO enrichment analysis showed that U-box proteins were mainly related to ubiquitination and modification in cells, followed by phosphorylation. Sixty-two genes were enriched in ubiquitination and metabolism (protein metabolic, primary metabolic, macromolecule metabolic, and cellular metabolic processes) and ligase activity, and 13 genes were enriched in phosphorylation. Six genes were enriched in response to stress and stimuli (Figure 6 and Supplementary Table 4).

### Expression characteristics of sorghum *U-box* genes

**FIGURE 7:**
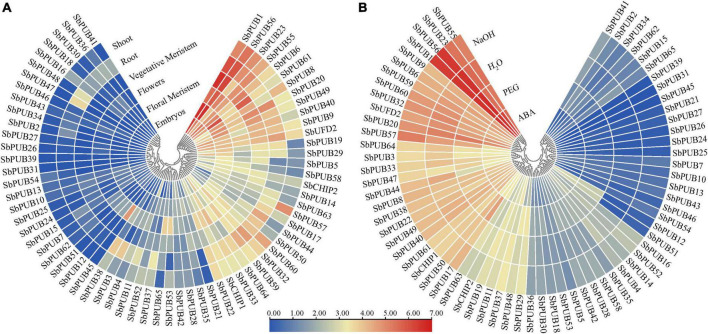
Heatmap of tissue-specific expression and abiotic stress expression of sorghum *U-box* genes. **(A)** Expression of *U-box* genes in different tissues. **(B)** Expression of *U-box* genes under the influence of NaOH, ABA, and PEG. The color bar represents the log2 expression level of each gene (FPKM, fragments per kilobase of exon per million fragments mapped). Color bar annotation is included at the top of the image. The heatmap is colored according to expression values, with blue, yellow, and red representing at low, medium, and high transcription abundance, respectively.

In order to explore the expression characteristics of *U-box* genes in sorghum, the expression level data were searched against the sorghum database. As shown in Figure 7, expression levels of *SbPUBs* in drought and osmotic responses were significantly different. In tissue-specific expression profiling, most *SbPUB* genes displayed tissue and organ expression specificity, especially in roots, meristems, embryos and other tissues associated with vigorous growth. The expression levels of *SbPUBs* and *SbCHIPs* were higher in most tissues, while those of *UFD2* genes were slightly increased in roots. The expression patterns of *PUB* genes could be divided into two categories; one group with low expression in the roots, meristems, embryos and other tissues (including the 24 *PUB* genes *PUB1/6/8/9/17/19/22/33/44/49/50/56/61/63/64*), and the other group was highly expressed in the roots, meristems, embryos and other tissues (including 41 *PUB* genes). The first group of *PUB* genes could be divided into three categories; Stem-specific expression enrichment (*PUB8/57/60*), root specific expression enrichment (*PUB17/20/44/50/55/59*), and flower and meristem specific expression enrichment (*PUB1/6/9/22/23/33/40/56/63/64*). The second group of *PUB* genes could also be divided into three categories; Root-specific expression enrichment (*PUB3/4/11/37/47/48*), floral and meristem specific expression enrichment (*PUB5/14/45/53/58/65*), and no significant expression (another 29 *U-box* genes; Figure 7A and Supplementary Table 5).

Regarding stress-related expression, *SbPUB* genes exhibited different expression characteristics when treated with PEG, ABA, and NaOH, distributing into three main types. Type I in which expression was inhibited (ABA response inhibition = *PUB2/15/21/27/29/30/34/36/41/44/46/48/50/54/57/60/64/65*, PEG response inhibition = *PUB15/41/44/65*, NaOH response inhibition = *PUB65*). Type II in which expression was promoted (ABA response upregulation = *PUB7/10/12/23/51*, PEG response upregulation = *PUB2/12/13/17/18/21/24/25/26/34/35/39/46/54/62*, NaOH response upregulation = *PUB12/51/62*), and type III with no significant expression (other *U-box* genes; Figure 7B and Supplementary Table 5).

### Co-regulatory networks involving sorghum *U-box* genes

**FIGURE 8:**
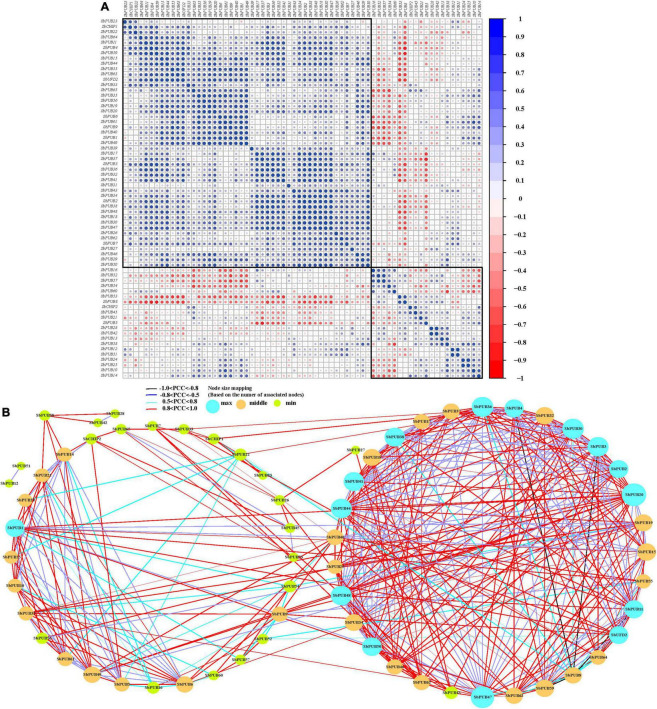
Correlation and co-regulation network of sorghum *U-box* genes. **(A)** Correlation analysis of *U-box* genes conducted by Pearson correlation coefficients (PCCs). Correlations are represented by the size and color of the circles. Heatmap labels indicate PCC values. **(B)** Co-regulatory network of sorghum *U-box* genes generated based on gene pairs with significant PCCs (*p*-value ≤ 0.05). The strength of correlations between gene pairs is marked at the bottom using different colored lines.

Based on the FPKM method (Tian et al., 2016), the PCC of *U-box* gene expression levels in *S. bicolor* and *A. thaliana* was calculated, and a co-regulatory network was constructed (Zhao et al., 2019). The results showed that ∼50% *U-box* genes were positively correlated. Additionally, the same gene type was mostly correlated, similar to those of *A. thaliana* (Figure 8A, Supplementary Figure 1, and Supplementary Table 6).

All significant PCCs (*p*-value ≤ 1e−5 and PCC >0.5) of *U-box* genes were extracted and Cytoscape was used to construct a co-regulation network. The co-regulation network of *S. bicolor* consists of 65 nodes and 406 edges (not including *SbPUB13*/*SbPUB31*/*SbPUB39*; Figure 8B). The results showed that the *U-box* genes in *S. bicolor* and *A. thaliana* were mainly positively correlated (1 > PCC > 0). Specifically, there were 123 strong positive correlations (0.8 < PCC < 1) and four strong negative correlations (−1 < PCC ≤ 0.8) in sorghum: *SbPUB3*/*SbPUB8*, *SbPUB4*/*SbPUB8*, *SbPUB11*/*SbPUB8*, and *SbPUB57*/*SbPUB8* (Figure 8). Meanwhile, there were 110 strong positive associations but no negative associations in *A. thaliana* (Supplementary Figure 1). In addition, there were weak negative correlations (−0.8 < PCC < 0.5) between *S. bicolor* and *A. thaliana U-box* genes (25.37 and 20.86%, respectively), and a dominant weak positive correlation (0.5 < PCC < 0.8) accounting for 45.4 and 43.35%, respectively (Supplementary Table 6).

### Polyethylene glycol and abscisic acid response characteristics of *U-box* genes determined by real-time quantitative PCR

To explore the key roles of the *U-box* gene family in the resistance process in sorghum, RT-qPCR was employed to assess the impact of plant in stress conditions. Figure 5 shows that drought response elements and ABA response elements were enriched in some genes, indicating that they may be stress tolerance genes (for those with >3 ABA response elements and drought response elements). Meanwhile, Figure 6 shows that many sorghum *U-box* genes exhibited obvious responses to ABA and PEG. Overall, these sets of results suggest that these genes may have important function for sorghum drought stress tolerance.

**FIGURE 9:**
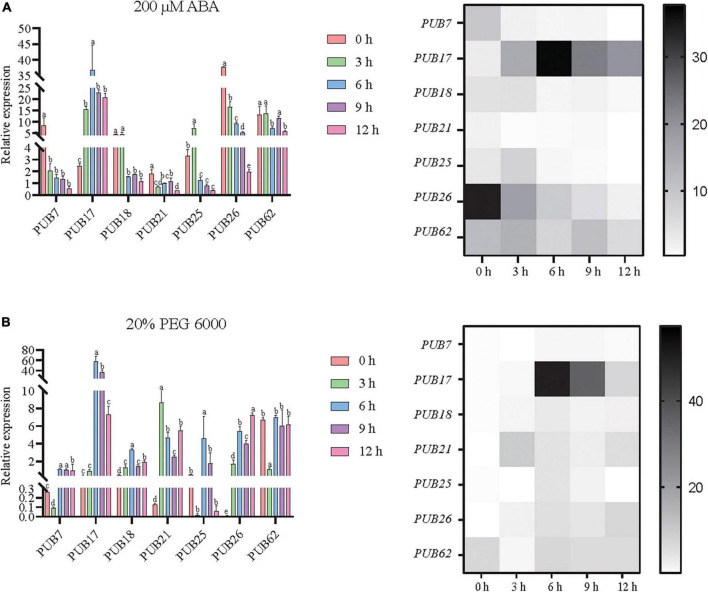
Potential responses of seven sorghum *U-box* genes to drought. All seven selected sorghum *PUB* genes (*SbPUB7*, *SbPUB17*, *SbPUB18*, *SbPUB21*, *SbPUB25*, *SbPUB26*, and *SbPUB62*) showed obvious expression differences under different conditions in heatmaps, and contain numerous drought elements. **(A)** Expression patterns of these genes in response to ABA-induced physiological drought. **(B)** Expression patterns of these genes in response to physiological drought induced by PEG 6000. Different lowercase letters indicate a significant difference determined by the Duncans new multiple range test (*P*-value < 0.05).

Seven *U-box* genes (SbPUB7/17/18/21/25/26/62) were characterized by high responses (>1.5-fold variation in expression) and abundant elements (>3 ABA and drought elements). Therefore, we quantitatively assessed these potential drought-regulated *U-box* genes. The results showed that the expression levels of these *PUB* genes were also significantly different according to RT-qPCR determination. Specifically, *SbPUB7* was significantly downregulated after ABA treatment. Expression of *SbPUB17* was significantly upregulated after ABA and PEG treatment, and the highest expression value was reached at 6 h. For *SbPUB18*, expression was significantly downregulated (6 h) after ABA treatment, but rapidly upregulated under PEG treatment. Similarly, *SbPUB21* was significantly downregulated by ABA treatment, but significantly upregulated by PEG treatment. After ABA treatment, expression of *SbPUB25* was first significantly upregulated then significantly downregulated to below the initial level, while expression of *SbPUB25* was significantly upregulated following PEG treatment at 6 h. Expression of *SbPUB26* was significantly downregulated after ABA treatment, and the expression level was significantly decreased with the extension of ABA treatment time, whereas expression of *SbPUB26* was significantly upregulated under PEG treatment. *SbPUB62* was significantly downregulated following ABA treatment at 6 and 12 h, and following PEG treatment at 3 h (Figure 9). These results indicate that there were differences in the response patterns of *U-box* genes to drought stress in sorghum, and there were significant differences in the response patterns of *U-box* genes to physiological drought induced by different conditions in sorghum. Most sorghum *U-box* genes were sensitive to ABA-induced physiological drought, but they were also linked to resistance to PEG-induced physiological drought. Interestingly, *SbPUB17* was upregulated under drought stress, while *SbPUB62* was downregulated.

### Gibberellin and methyl jasmonate response characteristics of *U-box* genes determined by real-time quantitative PCR

**FIGURE 10:**
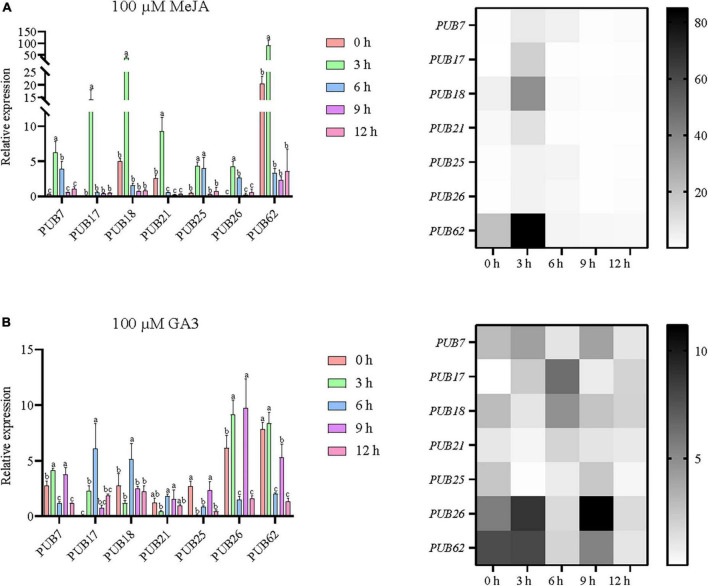
Potential responses of the seven sorghum *U-box* genes to methyl jasmonate **(A)** and gibberellin **(B)**. All seven selected sorghum *PUB* genes (*SbPUB7*, *SbPUB17*, *SbPUB18*, *SbPUB21*, *SbPUB25*, *SbPUB26*, and *SbPUB62*) show obvious expression differences under different conditions in heatmaps, and contain numerous JA/GA3 response elements. Different lowercase letters indicate a significant difference determined by the Duncans new multiple range test (*P*-value < 0.05).

As shown in Figure 5, the promoter regions of these *PUB* genes were rich in hormone response elements, especially those of JA and GA. In order to explore the response characteristics of sorghum *PUBs* under JA and GA, we further studied the previously selected seven genes by RT-qPCR. The results showed that all seven genes were upregulated in response to MeJA and GA3 treatment over a period (Figure 10).

Under MeJA treatment, expression of all seven sorghum *PUBs* was upregulated rapidly, and the expression level reached a peak at 3 h. Under longer treatment, expression of these genes dropped to initial levels or below. Specifically, *SbPUB7*, *SbPUB25*, and *SbPUB26* were significantly upregulated after 3 and 6 h of treatment then fell to the initial level after 9 h of treatment. Both *SbPUB17* and *SbPUB18* genes were significantly upregulated only after 3 h compared with initial levels, and expression then dropped to the initial level after 6 h of treatment. Both *SbPUB21* and *SbPUB62* genes were significantly upregulated after 3 h of treatment compared with the initial level, but dropped to below the initial level after 6 h of treatment (Figure 10A).

Under GA3 treatment, the expression patterns of the seven sorghum *PUBs* were significantly altered. In general, with the extension of treatment time, expression levels were first upregulated then downregulated. Specifically, expression levels of *SbPUB7*, *SbPUB25*, *SbPUB26*, and *SbPUB62* were significantly reduced at 6 and 12 h. The highest expression levels for *SbPUB17*, *SbPUB18*, and *SbPUB21* were obtained after continuous treatment for 6 h. In particular, the initial expression level of *SbPUB17* was extremely low, while the expression level was higher after GA3 treatment (Figure 10B).

## Discussion

Ubiquitylation, one of the most important modification types in plants, mediates the degradation of plant proteins and the process of protein repair. Most members of the *U-box* gene family are ligases, mediating the ubiquitination modification of protein, which enables ubiquitin to be combined with substrates to manipulate various protein activities (Azevedo et al., 2001; Monaghan et al., 2009; Wang et al., 2015; You and He, 2016; Qin et al., 2020). It has been reported that the expression of *U-box* genes can affect their functions both in plant development and stress conditions (Hu et al., 2018, 2019; Lu et al., 2020; Sharma and Taganna, 2020; Wang J. et al., 2020; Wang N. et al., 2020).

In the present study, we identified 68 *U-box* genes (including 1 *UFD2* and 2 *CHIP* genes) in *S. bicolor*. Additionally, CHIPs, UFD2, and U-box + WD40 branches were found to differ from other *U-box* genes in their characteristic sequence regions, at both protein and gene structure levels. U-box only and U-box + ARM branches have a relatively simple structure, consistent with the conclusions of previous studies. Most of the *U-box* gene families constitute about 50-80 members, such as rice, tomato, citrus, and *Arabidopsis*. Some plants have nearly 100 *U-box* genes (bananas = 91, cabbages = 101, and soybeans = 127). There are 93–208 *U-box* genes in cotton and 99 in cabbage, most within 6–8 in terms of sub-branch classification, such as apple, banana, cotton, and cabbage. Some contain more than 10, such as Chinese cabbage (Azevedo et al., 2001; Li et al., 2017; Hu et al., 2018; Chen X. X. et al., 2020; Lu et al., 2020; Qin et al., 2020; Rennie et al., 2020; Tang et al., 2021).

In this study, we could speculate that in the process of *U-box* gene cluster expansion, the original cluster contained only *U-box* genes with a few introns, from which substructures became embedded and shuffled, and mutations led to WD-40, ARM, TPR, and DWNN structures gradually appearing. Furthermore, *U-box* genes form large gene clusters at specific ends of chromosomes, and this cluster arrangement is also reflected in other plants (Qin et al., 2020; Wang N. et al., 2020). These gene clusters are likely to have occurred in recent years, because *CHIP* and some *PUB* genes are not only distributed at the beginning of chromosomes, and the beginning of chromosomes contain no clusters. We speculate that these genes differentiated early, and the beginning of chromosomes was not conducive to the enrichment of *U-box* genes. In addition, we only found one tandem repeat gene pair, namely *SbPUB26*/*SbPUB27*, which indicates that *U-box* genes only underwent one tandem repeat recently, hence we speculate that differentiation was minimal.

Collinearity analysis demonstrated that 9 arabidopsis *U-box* genes, 46 rice *U-box* genes, 59 maize *U-box* genes and 10, 46, and 45 sorghum *U-box* genes formed 14, 66, and 82 collinearity gene pairs, respectively. In addition, analysis of homologous *U-box* genes suggested that a large number of genes were derived from the differentiation of monocotyledons, such as *SbCHIP2*. We speculated that numerous *U-box* genes may exist in early sorghum plants, principally on chromosomes 1 and 4. During the course of evolution, a few *U-box* genes underwent various repetitions (e.g., fragment repetition, transposition, and tandem duplication) (Tan et al., 2016; Jiang et al., 2019; Vicient and Casacuberta, 2020; Wang J. et al., 2020; Wang N. et al., 2020; Xiao et al., 2020), resulting in the characteristic gene distribution (i.e., genes are widely distributed except on chromosome 5).

Furthermore, numerous tissue-specific elements were found in the *U-box* promoter regions. Analysis of expression characteristics revealed that *U-box* genes displayed obvious tissue specificity, with highest expression levels in roots, flowers, meristem, and stems. Similarly, obvious tissue expression was evident for *U-box* genes in bananas, with high expression levels in roots (Hu et al., 2018). In soybean, selective tissue expression was also observed in the roots of young tissues and flowers (Wang N. et al., 2020). Similar expression patterns were also found in cotton, cabbage, and other plants (Wang et al., 2015; Song et al., 2017; Hu et al., 2018; Sharma and Taganna, 2020). In the sorghum *U-box* promoter regions, we identified numerous abiotic response elements, especially those related to drought, ABA, JA, and GA responses, and this was confirmed by RT-qPCR (Figures 5, 7, 9, 10). Previous studies have shown that many *U-box* genes mediate drought tolerance in plants through multiple pathways. For example, *OsPUB67*, *AtPUB11*, *AtPUB18*, *AtPUB19*, *AtPUB22*, and *AtPUB23* are involved in drought tolerance in an ABA-dependent manner (Chen X. X. et al., 2020; Qin et al., 2020). Drought-responsive genes were significantly upregulated in *GmPUB6*-overexpressing plants, indicating that this gene mediates osmotic stress and ABA signaling pathways to enhance drought tolerance in plants (Wang N. et al., 2020). In response to physiological drought, the *SbU-box* genes displayed different expression pattern under PEG and ABA treatments, and the responses could be mainly divided into three types: type I (suppressed expression), type II (upregulated expression), and type III (no significant change in expression). In our study, seven genes (*PUB7/17/18/21/25/26/62*) differentially expressed at different time points following abiotic stress were examined. Most *U-box* genes in sorghum are physiological drought-sensitive genes induced by ABA, but some are physiological drought resistance genes induced by PEG. Interestingly, *SbPUB17* was upregulated following physiological drought caused by two different reagents, while *SbPUB62* was downregulated following physiological drought caused by two different reagents. Previous studies have shown that many *U-box* genes can respond to JA and GA hormones, thereby promoting plant stress resistance. For example, *AtPUB10* and *StPUB17* are induced by JA, and they promote the stability of the JA hormone pathway, whereas *StPHOR1* is induced by GA, and this mediates the GA hormone pathway (Amador et al., 2001). In our study, under MeJA treatment, expression of the seven *PUB* genes in sorghum was rapidly upregulated, and expression peaked at 3 h. Over a longer period, expression of these genes dropped to initial levels or lower. Under GA3 treatment, the expression patterns of the seven sorghum *PUB* genes were changed significantly. In general, with the extension of treatment time, expression levels were typically upregulated initially then downregulated. In particular, the initial expression level of *SbPUB17* was very low, and this increased following GA3 treatment.

The co-regulatory network and correlations between plant *U-box* genes were similar to those of most plant gene families. Among the same groups, the co-regulatory network and expression correlations were relatively high and mainly positive (Lv et al., 2017; Zhao et al., 2019; Zhang et al., 2020). However, we only carried out genome-wide identification and structural analysis of sorghum *U-box* genes, hence the detailed involvement of *U-box* genes in plant development remains unclear. Previous studies have shown that *U-box* genes may be associated with cold stress (Hu et al., 2018; Lu et al., 2020). Additionally, functional differentiation and functional responses have been linked to sorghum *U-box* genes, but the details remain to be verified.

## Conclusion

In this study, we identified and characterized 68 sorghum *U-box* genes, which are distributed across all chromosomes except chromosome 5, and divided into eight subclasses. The gene co-regulation network and expression correlations were relatively high and mainly positive. RNA-seq and RT-qPCR experiments showed that the sorghum *U-box* genes could be tissue-specific, and are involved in responses to various stresses. The results provide a list of potential genes involved in the sorghum ubiquitination system, and expand our understanding of the evolution of the sorghum genome.

## Data availability statement

The datasets presented in this study can be found in online repositories. The names of the repository/repositories and accession number(s) can be found in the article/Supplementary material.

## Author contributions

YF, JJ, and QD conducted the experiment. YF wrote the manuscript. ZY and XL directed the experiments and participated in the revision of the manuscript. YF, XH, QY, DZ, XL, and XX completed the revision of the manuscript. All authors contributed to the article and approved the submitted version.

## Footnotes

1
http://plants.ensembl.org/

2
http://meme-suite.org/tools/meme

3
https://web.expasy.org/

4
https://itol.embl.de/

5
https://orthovenn2.bioinfotoolkits.net/

6
http://bioinfo.cau.edu.cn/agriGO/index.php

7
http://www.aipufu.com/

8
http://bioinformatics.psb.ugent.be/webtools/plantcare/html/

9
https://www.ncbi.nlm.nih.gov/sra/

10
https://www.ebi.ac.uk/gxa/home

## Abbreviations

PUB: plant U-box protein
CHIP: heat shock protein 70-interacting protein
UFD2: Ub fusion degradation 2
PEG: polyethylene glycol
ABA: abscisic acid
GA: gibberellin
MeJA: methyl jasmonate
HMM: hidden Markov model
PI: isoelectric point
FPKM: fragments per kilobase of exon per million fragments mapped
PCC: Pearson correlation coefficient
NJ: neighbor-joining
RT-qPCR: real-time quantitative PCR
PTM: post-translational modification
UAE: ubiquitin-activating enzyme
UBC: ubiquitin-conjugating enzyme
HECT: homologous to the E6-AP carboxy terminus
CRL: cullin-ring ubiquitin ligase
GO: gene ontology
BBX: novel B-box protein
AIRP: abscisic acid (ABA)-insensitive RING protein
LRR1: leucine rich repeat protein 1
kin7: Kinase 7.
