# Supplementary material for: Identification, characterization, and expression profiling of the putative U-box E3 ubiquitin ligase gene family in Sorghum bicolor
Source: Front Microbiol. 2022 Sep 15;13:942302. doi: 10.3389/fmicb.2022.942302 (PMC9520534; doi:10.3389/fmicb.2022.942302)
Supplement: Supplementary file 1 [file Figure S1.pdf › Supplementary Material/Figure S1.pdf]

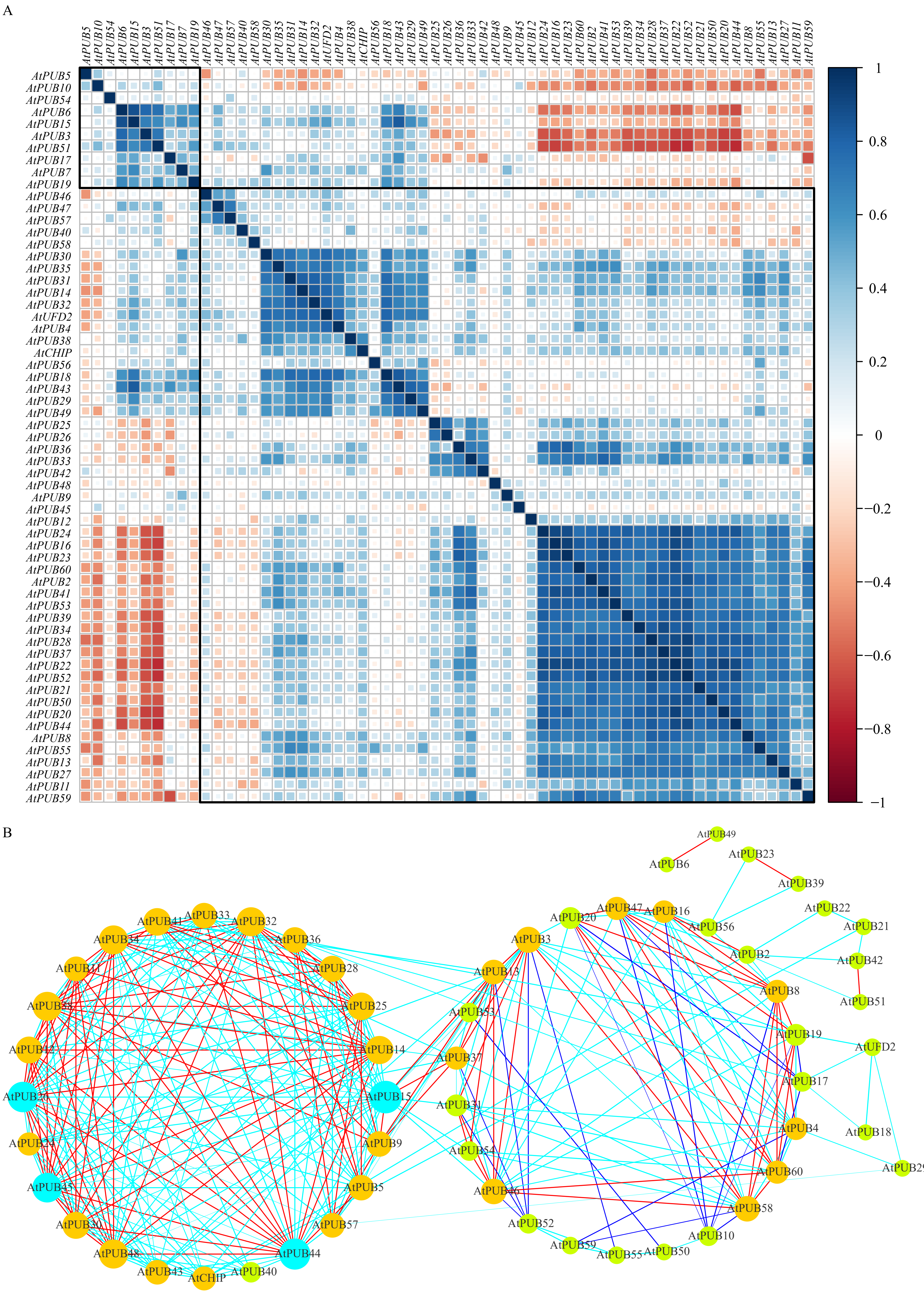
A
B
1
0.8
0.6
0.4
0.2
0
−0.2
−0.4
−0.6
−0.8
−1
AtPUB5
AtPUB10
AtPUB54
AtPUB6
AtPUB15
AtPUB3
AtPUB51
AtPUB17
AtPUB7
AtPUB19
AtPUB46
AtPUB47
AtPUB57
AtPUB40
AtPUB58
AtPUB30
AtPUB35
AtPUB31
AtPUB14
AtPUB32
AtUFD2
AtPUB4
AtPUB38
AtCHIP
AtPUB56
AtPUB18
AtPUB43
AtPUB29
AtPUB49
AtPUB25
AtPUB26
AtPUB36
AtPUB33
AtPUB42
AtPUB48
AtPUB9
AtPUB45
AtPUB12
AtPUB24
AtPUB16
AtPUB23
AtPUB60
AtPUB2
AtPUB41
AtPUB53
AtPUB39
AtPUB34
AtPUB28
AtPUB37
AtPUB22
AtPUB52
AtPUB21
AtPUB50
AtPUB20
AtPUB44
AtPUB8
AtPUB55
AtPUB13
AtPUB27
AtPUB11
AtPUB59
